# Supplementary material for: Are carbon emissions trading and green financial instruments synergistic? -Comprehensive quantitative research based on content analysis
Source: PLoS One. 2024 Mar 7;19(3):e0298601. doi: 10.1371/journal.pone.0298601 (PMC10919601; doi:10.1371/journal.pone.0298601)
Supplement: S1 Data — (ZIP) [file pone.0298601.s001.zip › Data/地级市Z_F工作报告.zip!/║╙─╧/σo╤⌠╩╨/└┤╫╘╣1⁄2╓┌║┼í╛┬φ┐╦╩2╛▌═°í┐_2017.docx]

各位代表：

现在，我代表市人民政府，向大会报告工作，请予审议，并请各位政协委员和其他列席人士提出意见。

一、2016年工作回顾

2016年是濮阳蓄势转型、奋力赶超的一年。一年来，在省委、省政府和市委的坚强领导下，全市上下全面落实党的十八大和十八大以来历次中央全会精神，深入贯彻习近平总书记系列重要讲话精神，统筹推进“五位一体”总体布局，协调推进“四个全面”战略布局，坚持新发展理念，主动适应经济发展新常态，以创新发展、转型发展、赶超发展为引领，着力推进供给侧结构性改革，稳增长保态势，调结构促转型，抓改革增活力，夯基础创优势，办实事惠民生，拼搏进取、克难攻坚，实现了经济总量、人均数量、发展质量“三量齐升”，圆满完成了市七届人大三次会议确定的目标任务，实现了“十三五”良好开局。

（一）赶超发展态势良好

一年来，我们沉着应对经济下行压力，着力扩大有效投资，重点实施了341个省市重点项目，完成投资1002亿元；推出了降本增效“三十条”、支持民营经济发展“十五条”等多项政策措施，实现了经济平稳较快增长。2016年，预计全市生产总值完成1424亿元，增长8.7%，增速居全省第6位；规模以上工业增加值完成707亿元，增长9.1%，居全省第3位；固定资产投资1523亿元，增长16.7%，居全省第5位；社会消费品零售总额530亿元，增长12.5%，居全省第2位；一般公共预算收入72.16亿元，为调整预算后的102.2%；一般公共预算支出221.57亿元，为调整预算后的97.9%；居民人均可支配收入16650元，增长8.5%，居全省第1位。主要经济指标增速位居全省第一方阵，赶超发展步履坚实。

（二）转型升级步伐加快

工业持续优化升级。化工产业高端化发展步伐加快，以惠成电子、蔚林化工等为代表的化工新材料和专用化学品企业迅速成长。高新技术产业和战略性新兴产业增长28.7%，高出全市工业增速19.6个百分点。产业集聚度不断提高，全市产业集聚区完成投资1034亿元，增长34.5%；完成主营业务收入1784亿元，增长17.8%。濮阳县、清丰县产业集聚区晋级一星，濮阳市化工专业园区晋位省级。民营企业不断壮大，濮耐等4家企业进入省民营企业100强。盛源等7家企业进入省民营企业制造业100强，数量居全省第5位。

服务业发展迅速。全年完成投资666亿元，增长26.1%。新增规模以上企业240家。第三产业增长10.5%。三产比重提高0.9个百分点，有力拉动了经济增长。商贸物流业不断壮大，正大商贸物流、上亿商业广场等项目投入运营，恒丰城市广场、恒大悦龙台城市综合体等项目加快建设。旅游业增长迅速，水库龙山、濮阳国际杂技文化产业园、东北庄旅游景区、单拐红色旅游景区等成为旅游新热点，全市旅游业收入16亿元，增长82%。金融业发展实现新突破，农信社改制加速推进，郑州银行、中原农业保险股份有限公司入驻濮阳，新开设村镇银行支行5家。辖内银行业金融机构各项贷款余额636亿元，增长17%，增速居全省第5位。信息产业和电子商务发展势头良好，大数据智慧生态园已有11家企业注册入园。全市电商企业达到169家，交易额240亿元，增长20%。开发区、清丰县、南乐县、台前县电商产业园快速发展，范县、濮阳县分别被新确定为国家、省级电子商务进农村综合示范县。服务业“两区”加快建设，实现主营业务收入110亿元，税收8.2亿元。华龙区特色商业区荣获省先进服务业“两区”称号。

农业增收增效。持续稳定耕地面积、粮食生产能力，粮食生产再获丰收。流转土地15万亩，新增现代农业园区39个。国家、省级农业产业化龙头企业达到29家。新增农民专业合作社309家，国家级示范社达到29家。农产品加工企业实现销售收入353亿元，增长10%。农作物耕种收机械化率达到82%。彭楼等大中型灌区续建配套与节水改造任务圆满完成，新增有效灌溉面积2.9万亩、节水面积10.6万亩。

（三）基础能力显著提升

交通体系建设取得重大突破。晋豫鲁铁路濮阳站开通客运，结束了我市不通国铁客运的历史。京九客专在濮阳过境设站大势已定。郑濮济高铁郑州至濮阳段开工建设，濮阳人乘高铁出行，将梦想成真。范台梁高速开工并快速推进建设，台前县孙口黄河大桥及引线工程复工建设。新建改造G341、S307等干线公路115公里，通车62公里。干线公路通车总里程达到615公里。新完成改建农村公路618公里。

新型化工基地加快布局。高点定位，顶层设计，规划建设的60平方公里新型化工基地，获得了省政府支持和行业认可，为我市产业转型、接续发展提供了战略平台。丰利石化原油进口使用资质通过国家专家组核查评估，昊华骏化100万吨醋酸制乙醇项目开工建设，中国机械工业集团聚碳酸酯项目签约入园，国家石油化工应急救援（濮阳）中心获批。

资源能源配置能力不断提高。油地战略合作持续深化，双方协同推进了炼化一体化等重大产业项目。文23地下储气库先导工程完工，中原油田至开封输气管道建成投用，龙丰2×60万千瓦机组、引黄入冀补淀工程等加快建设，晶科光伏等5个项目并网发电。新建通讯铁塔244座，改建878座，共享率达到83.5%。城镇实现光纤、4G网络全覆盖，农村覆盖90%以上。

（四）改革创新活力显现

重点领域改革取得新进展。加快“放、管、服”改革，下放行政审批和管理权限39项，清理规范中介服务事项414项；全市商事制度实行“五证合一”。深化投融资体制改革，市投资集团融资能力显著增强，总资产规模达到260亿元，全年完成融资66.7亿元，首发私募公司债15亿元。获准注册银行间市场融资工具10亿元。实施了第二污水处理厂二期等13个PPP项目，总投资131.1亿元。佳化能源等4家企业在“新三板”挂牌交易。31家国企改革和“四供一业”移交扎实推进。农用地、集体建设用地、宅基地确权颁证基本完成，农村产权制度改革走在全省前列。深化新型城镇化综合改革，“三改一化”试点扎实推进。持续实施“农民安家贷”“学位券”等房地产新政，商品房库存去化周期处于合理区间，濮阳经验在全国推广。医疗卫生、养老保险、教育等改革都取得新成效。

科技创新能力进一步提高。新增高新技术企业8家、省“科技小巨人”企业5家、科技型中小企业50家。新增省市级工程技术研究中心20家、市级重点实验室13家。濮阳光电产业技术研究院等3家机构列入省新型研发机构。建成盛源能源院士工作站，其绿色烷基化技术打破国外技术垄断，达到国际先进水平。濮耐新型喷射浇注料填补国内空白。柔性引进人才1036人。新增有效发明专利111件，万人有效发明专利拥有量1.4件，增长19.8%。诚农蔬菜公司星创天地被认定为国家级农业众创空间，濮阳电商园被认定为省级众创空间，全市新增市级众创空间10家，促进了大众创业、万众创新。

（五）开放招商成果丰硕

一批重大项目落户濮阳。走出去，赴长三角、珠三角、京津冀等地开展专业招商，与力帆、华谊、华强、绿地、四川中大及德国巴斯夫、美国PPG等80余家国内外行业龙头开展了深层对接，与保利协鑫、中建材、美涂士、北京红狮、天津大田等40余家知名企业签订了战略合作协议；请进来，成功举办中国（濮阳）石化产业精细化发展大会、全国电子化学品产业（濮阳）发展论坛、第三届中原（濮阳）油气技术装备展览会、中国濮阳涂料产业发展研讨会等8次重大专题招商活动。以市场换产业，引入了东方雨虹、德力西电气、森源重工等一批产业项目。中机公司3D打印材料产业园、蓝星丁二烯抽提等372个超亿元项目成功签约。96家京津冀家居企业落户清丰县。王老吉中部创新基地、嘉远新能源电动汽车等304个超亿元项目开工建设。2016年，全市利用省外资金210亿元、利用境外资金6.33亿美元，分别增长8%、10%，增速分别居全省第1位、第3位。

开放水平不断提升。着力抓好开放平台建设，国检濮阳办事处业务中心建成投用。郑州海关濮阳监管办事机构即将获准。跨境电子商务示范园区加快建设。全市外贸出口总额7.45亿美元，增长5.7%。服务贸易出口1.47亿美元，居全省第2位。中原石油工程公司等3家企业进入全省出口企业50强。省杂技集团收购美国布兰森大剧院，企业“走出去”迈出新步伐。

（六）城乡建设管理扎实推进

坚持规划引领，完成了濮阳市第四轮城市总体规划修编纲要，启动县城总体规划修编。加强基础设施建设，第三水厂投入运营，市城区新铺设雨污水管线27公里，扩建了第一、第二污水处理厂，新建改造金堤路、香山路等18条道路，优化调整公交线路6条、新增营运车辆200辆，开工建设了6个公共停车场。公共自行车租赁系统正式运营，首期投放自行车2000辆。建成市图书馆、市工人文化宫。城市精细化管理取得明显成效。县城基础设施和公共服务能力明显提升。台前县创建成为省级园林县城。大力推进棚户区改造，当年实施东干城等42个项目，其中，15个项目完成拆迁，8个项目建成回迁。新谋划纳入省棚改计划项目20个，争取补助资金3.5亿元。综合提升小城镇，文留镇等5个镇创建成国家卫生镇，濮城镇创建成全国文明镇，元村镇被评为国家级生态镇。坚持“一基本、两牵动、三保障”，全市城镇化率达到42%。统筹推进美丽乡村建设，突出产业培育、道路畅通、环境美化、文化建设等，部分乡村面貌大为改观。南乐县、清丰县实现城乡垃圾一体化处理，高堡村等10个村被列入省美丽乡村建设试点。

（七）生态建设力度加大

全力打好大气污染防治攻坚战。上下联动，压实责任，坚持“六控”并举，深入开展污染围城和“小散乱差”企业治理，加强挥发性有机物治理，实施市城区烟花爆竹禁放，启动重污染天气应急管控措施，严查环保违法违规行为，全市空气质量特别是7月4日打响大气污染防治攻坚战以来明显改善，PM10、PM2.5分别较上年同期下降18.4、12.7个百分点。全年优良天数181天。

切实加大环境整治建设力度。强化河道监管责任，率先推行“河长制”。完成了为期三年的濮水河、新老马颊河市城区段岸堤、道路提升任务。濮上河竣工通水。我市成功获批省海绵城市试点市，完成项目36个。开展国家森林城市创建，植树1423万株，完成廊道绿化、环城林系建设900公里，实施水库龙山绿化、城市绿地游园和县城绿化美化。全市新增绿化面积130万平方米，森林覆盖率达到27.5%。万元生产总值能耗下降5个百分点。

（八）人民生活持续改善

2016年，十项重点民生工程全面完成，全市财政的民生项目支出172.84亿元，占一般公共预算支出的78%。

脱贫攻坚成效明显。开展“双联四推六覆盖”活动，首创智慧精准扶贫系统，实施1 + 5产业扶贫、1 + 2社会保障扶贫等重大工程，扶贫工作成效显著。黄河滩区第一批迁建试点工程完工，部分群众喜迁新居，第二批迁建试点加快建设。4.1万人实现脱贫，超额完成省定目标。

就业社保不断强化。城镇新增就业6.1万人，3.3万失业与就业困难人员上岗就业。新增农村劳动力转移就业2.6万人。支持小微企业发展，发放创业担保贷款6.95亿元，吸纳1.9万人就业。城市、农村低保年补助标准分别增加180元、204元，五保对象年补助标准增加200元。企业退休人员基本养老金实现“十二年连增”。城镇医保和新农合年人均补助标准提至420元。发放失业保险金1.1亿元。建成农村幸福院1173个、社区日间照料中心15个。

社会事业全面发展。实施中小学校舍安全工程项目53个，改善125所义务教育薄弱学校办学条件，农村成建制学校全部达到省定办学标准。深入开展社会主义核心价值观教育，继续开展全民敬业活动。实施文化惠民工程，市图书馆20个社区分馆和一批农村综合文化服务中心建成投用，组织文艺下农村下基层活动3.6万场次，丰富了群众文化生活。承办了第七届黄河戏曲节，新编历史剧《大清诤臣窦光鼐》荣获金奖。举办了全国青少年中国式摔跤锦标赛等全国性赛事，全民健身活动蓬勃开展。市残疾人综合服务中心、市民综合活动中心加快建设。改造提升14所社区卫生服务中心，圆满完成乡村医疗机构标准化建设任务。2016年，通过加大投入，调整水源，市城区居民全都喝上了纯净的丹江水。

社会大局和谐稳定。安全生产形势总体平稳，食品药品安全形势持续向好。平安濮阳、法治濮阳建设扎实推进，社会大局保持和谐稳定。人民群众对政法机关综合执法满意度、公众安全感连续四年荣获全省“双第一”。

（九）政府自身建设不断加强

坚持依法行政，完善政府运行机制，规范行政权力，行政效能不断提升。认真执行人大及其常委会决议和决定，支持政协参政议政，做好人大议案、代表建议和政协提案办理工作。广泛听取民主党派、工商联、无党派人士意见，支持工会、共青团、妇联等人民团体开展工作，自觉接受各方监督。落实全面从严治党主体责任，深入开展“两学一做”学习教育，认真贯彻中央八项规定精神，整治懒政怠政、为官不为，干部作风进一步优化。强化行政监察、审计监督、投资监管，营造为民、务实、清廉的政务环境。

国防动员教育以及双拥工作扎实开展，驻濮解放军和武警部队在支持地方发展、维护稳定方面做出了积极贡献。统计、发展研究、民族宗教、外事侨务、机关事务、驻外联络、公积金管理、气象、防震减灾、人防、史志、档案等工作都取得了新成绩。

各位代表！一年来，全市上下凝心聚力、矢志赶超，克服了多重困难，取得了丰硕成果。成绩来之不易，这是省委、省政府和市委正确领导的结果，是市人大、市政协监督支持的结果，是全市人民团结奋进、担当奉献、不懈进取的结果。在此，我代表市政府，向工作在各条战线的工人、农民、知识分子、干部、人民解放军指战员、武警官兵、公安民警以及所有关心、支持濮阳发展的各界人士、社会各界朋友致以崇高的敬意和衷心的感谢！

同时，我们也清醒地认识到，经济社会发展中还存在一些突出问题：一是产业结构调整任务艰巨。接续产业发展不快，传统产业占比偏高，战略性新兴产业规模较小，现代服务业发展相对滞后，农业结构仍需优化。二是产业金融结合亟待加强。运用市场机制、金融手段推动产业发展的能力不强，一些可以市场化推进的项目进展不快。三是招商引资落地慢。全产业链招商、全要素配套保障、全过程跟踪服务的招商落地机制还不健全。四是个别领域改革力度需要进一步加大。对改革的大局意识和紧迫感认识不足。五是环境问题突出。与省定目标相比，我市PM10、优良天数还有差距。大气污染防治、水污染治理和农村环境整治仍需下大气力。六是发展环境还需进一步优化。一些部门效能不高，一些干部创新意识、担当意识不强，部分项目推进不快。对此，我们一定高度重视，认真加以解决。

二、2017年工作总体要求和主要目标

2017年是全面落实省十次党代会和市七次党代会精神的开局之年，也是深入贯彻中央重大战略部署、实施好“十三五”规划、致力濮阳赶超发展的关键一年。做好今年工作，意义重大。

综合分析，2017年，经济形势依然复杂严峻。在全球经济增长乏力、国内经济调整转型的大背景下，推动经济社会发展，我们承担着赶超发展与转型发展、与提质发展的艰巨任务，又面临着赶超发展与保护环境、与提升基础支撑能力的艰巨重担，挑战前所未有。同时，更要看到，国家已经确立了适应经济发展新常态、以新发展理念为指导、以供给侧结构性改革为主线的政策体系框架，经济政策更稳、更准、更活、更实、更托底，也更有操作性和可预期性；确立了稳中求进的工作总基调，经济长期向好的基本面没有变，经济增长有弹性、有空间、有余地，蕴含着机遇；国家和省一系列重大战略正在展开布局落地，在资源城市转型发展、新型城镇化建设、支持创新创业、提升社会保障能力等方面，都为濮阳带来机遇；国家将继续实施更加积极的财政政策和稳健的货币政策，在交通、水利、扶贫开发、环境治理、产业升级等方面继续加大投资力度，为我市补短板、创优势提供了政策机遇；国家产业政策更加精准，产业转移及化工项目进园区，为我们招商引资，建设新型化工基地提供了千载难逢的好机遇。特别是市七次党代会规划了建设富裕文明和谐美丽新濮阳的宏伟目标，提出了创新发展、转型发展、赶超发展的战略任务，全市上下凝心聚力、奋发进取，积累了工作成果，积蓄了强大势能。

综合判断，机遇与挑战并存，机遇大于挑战。只要我们坚定信心，奋力拼搏，濮阳发展大有可为。

2017年政府工作的总体要求是：全面贯彻党的十八大和十八届三中、四中、五中、六中全会精神，更加紧密地团结在以习近平同志为核心的党中央周围，全面落实中央、省委、省政府和市委决策部署，统筹推进“五位一体”总体布局，协调推进“四个全面”战略布局，牢固树立和贯彻落实新发展理念，坚持稳中求进工作总基调，以供给侧结构性改革为主线，突出抓好“三去一降一补”，突出打好“四张牌”，着力创新发展，深化改革、扩大开放，培育增长新动能；着力转型发展，优化结构、提升层次，促进产业迈向中高端；着力赶超发展，推动增长、厚植优势，努力实现“三量齐升”；着力共享发展，改善民生，托住底线，让人民群众生活得更美好，加快建设富裕文明和谐美丽新濮阳，以优异成绩迎接党的十九大胜利召开。

2017年我市经济社会发展的主要预期目标是：生产总值增长8.5%以上；规模以上工业增加值增长9%；一般公共预算收入增长8%；固定资产投资增长15%以上；社会消费品零售总额增长12.5%；外贸进出口总额增长5%；城镇新增就业4万人；居民人均可支配收入增长8.5%；居民消费价格指数涨幅控制在3%左右；节能减排完成省定目标，环境质量持续改善。

实现上述预期目标，关键要坚持稳中求进工作总基调，按照打好“四张牌”的要求，在确保“稳增长”的前提下，各项工作都要突出“进”，突出奋发有为。当前和今后一个时期，我们要持续抓好“四大核心任务”：

一是加快产业结构优化升级。产业是一个地区的脊梁。濮阳是资源型城市，转型升级是突出任务；濮阳经济基础薄弱，赶超发展是历史使命。我们必须紧紧抓住转型升级这一突出任务不放松，必须紧紧抓住赶超发展这一长期任务不动摇，研究产业发展阶段性特征，遵循资源型城市产业升级规律，坚持转型发展与赶超发展并重、壮大规模与提高质量并重，在转型中快发展，在发展中提效益，闯出一条欠发达地区弯道超车、转型升级的新路子。

二是大力实施创新驱动发展。创新是引领发展的第一动力。我们必须认清经济新常态大逻辑，把握经济发展大趋势，学会市场运作新本领，用创新的理念谋划工作、推动发展，用创新的方法解决新常态下的矛盾和问题，用创新的思路汇聚生产要素，实现产业、资金、技术、人才与政策相适配的融合发展，努力打造区域性创新新高地。

三是强化基础能力建设。基础能力是一个地区的战略支撑。我们必须补足历史欠账，补齐发展短板，筑牢发展根基。坚持以项目建设为抓手，科学谋划项目，有效运作项目，加快实施项目，构建现代交通体系、现代物流体系、现代城镇体系、水和能源保障体系、生态保障体系、信息传输体系、公共服务体系、对外开放体系，厚植赶超发展新优势。

四是推进新型城镇化。城镇化是现代化的必由之路。城镇化承载工业、催化服务业、促进现代农业，具有拉动投资、扩大消费的双重功能。我们必须坚持以人的城镇化为核心，发挥产业拉动、户籍住房牵动、市场和资源带动、农村产权改革推动、社会保障托动等综合效应，提升城镇化水平，形成城乡协调发展新格局。

三、2017年重点工作

2017年，围绕上述“四大核心任务”，要重点抓好九方面工作。

（一）推动经济平稳较快增长

把握拉动增长的关键环节，抓住项目，做强实体，服务企业，优化环境，继续保持经济发展的良好态势。

1.抓项目扩大有效投资。着力实施好369个省、市重点项目，年内完成投资1012.7亿元以上。其中，新开工221个，续建143个，106个项目建成投产投用。加大项目推进力度，确保早开工、早建设、早见效。对接上级政策，争取更多项目纳入国家、省计划盘子。大力推行PPP模式，统筹规划、统筹建设、一体化运作，着力降成本、保质量。同时，注重创造消费新需求，培育消费新热点，充分发挥消费的拉动作用。

2.大力发展实体经济。把发展实体经济作为稳增长的“压舱石”，摆在更加突出的位置。着力为企业松绑降本、减负增效，确保国家、省和我市支持实体经济发展的系列政策措施落实到位。继续深化“惠企政策进千企”系列活动，搞好四项对接，按照企业需求，强化精准服务，壮大过桥资金、助保贷规模，帮助企业开拓市场，切实解决企业生产经营中的问题。积极争取国家、省专项基金，支持企业产品创新、服务创新、业态创新。搞好经济运行监测预警，及时发现和解决苗头性、倾向性问题，防止出现大的波动。壮大民营经济，落实民营经济“十五条”政策，实施“百强企业扶持计划”“中小企业成长计划”，支持企业通过技改扩能、兼并重组、资产扩股等形式发展壮大，催生一批高成长性企业。

3.保持房地产平稳健康发展。坚持多措并举，继续实行鼓励农民进城购房的政策，促进房地产投资稳定增长、销售平稳运行、价格基本稳定。统筹土地供应，优化供给结构，满足进城农民住房需求和城市居民改善性住房需求。抓住政策窗口期，打好棚户区改造攻坚战，全年新实施54个棚改项目，新建和货币化安置住房4.34万套，当年建成1万套。加强监管，规范房地产开发、销售、中介服务、物业管理等行为。

（二）加快产业转型升级

按照建设“四个强省”的要求，以提高质量效益为中心，以项目建设为抓手，重塑产业链、价值链，做强二产、做大三产、做优一产，积极培育网络经济，全面提升产业竞争力。

1.提升层次，做强工业。年内实施重点工业项目105个，计划完成投资316亿元。

改造提升传统优势产业。按照整合资源做大基础化工、延伸链条发展高端化工的思路，加快布局建设新型化工基地，完善管理体制机制，统筹做好土地、产业、搬迁安置、基础设施、公共服务等建设规划，开工建设一批道路、管网等基础设施项目。育龙头壮大基础化工，丰利石化扩能改造取得重大进展，昊华骏化醋酸制乙醇、蓝星丁二烯抽提等项目加快建设，谋划推进中原炼化一体化等重大项目。拉链条发展化工新材料和专用化学品，开工建设蔚林化工橡胶助剂产业园、3D打印新材料产业园等项目，加快建设惠成电子技改扩能、盛华德精细化工原料、东方雨虹涂料产业园等项目，建成富士康正一特殊材料一期、班德路化学加氢树脂、迈奇丁内酯等项目。加快推进开发区电子化学品园区建设。装备制造、家具制造、食品加工、羽绒及服饰加工等传统产业，通过技改扩能、引进合作、产品创新、品牌建设等途径，提升竞争力。重点实施华南重工高压电机、美松爱家环保包装、雪汇食品冷饮、鹏达羽绒等87个技改项目。加大新建项目推进力度，年内开工建设德众汽车产业园、奥斯玛电梯、南乐冷冻食品产业园等25个项目。加快推进华远达电气控制设备、浙江商会家居产业园等20个项目。建成德力西电气、慷达食品等24个项目。

加快培育发展战略性新兴产业。抓好国家生物基材料产业基地建设，完善产业链、创新链、资金链、政策链，推动集群集聚发展。年内建成L-乳酸及秸杆制乳酸一期、聚乳酸改性树脂扩建等项目。加快发展新能源产业，保利协鑫天然气分布式能源、华能濮阳县500MW风电等项目开工建设，润清风电场、华电光伏等4个项目年内实现并网发电。培育发展新能源汽车，开工建设爱蓝电动汽车、江苏新世纪低速电动车等项目，加快建设嘉远新能源电动汽车、汉阳电子汽车零部件等项目，建成天能电池三期工程，积极推进望联机械与东风二汽深度合作，研发专用车辆。培育发展节能环保、生物医药产业，加快建设明锐节能节水装置、雷神医疗、海王百草堂中医药产业园等项目。

2.聚焦重点，做大服务业。年内实施重点服务业项目58个，完成投资150亿元。

提升商贸物流业。加快推进宏进农副产品物流中心、机动车循环经济产业园、金宇国际商城等项目，建成投用豫鲁商贸物流港三期、工业园区华宏物流储运中心等项目。全力抓好市城区农贸市场建设三年（2016—2018）攻坚行动，加快农贸市场改造建设步伐。10月底前恢复中原市场、劳动市场2个市场的农贸区功能，并投入使用；年内完成波头集市场、东风市场等4个市场的改造提升；新建文明路市场、王助市场等5个市场。

壮大文化旅游业。依托我市独特的文化和生态资源，重点打造“游龙都、看杂技、赏黄河、品美食”特色旅游产品。开工建设中华龙源景区，叫响华夏龙都品牌。建成国际杂技文化产业园二期工程，完善提升东北庄杂技文化园区，开工建设东北庄野生动物园，推动杂技艺术学校开放办学，让濮阳杂技享誉中外。利用黄河生态优势，规划建设一批黄河湿地、黄河景区和水利景观，打造“五彩黄河”生态休闲旅游带。挖掘红色历史文化资源，推出红色旅游精品线路。推进滨河特色商业街、澶州州署复建等项目，大力推介濮阳，让文化旅游成为我市发展新亮点。

发展金融服务业。增强市投资集团融资能力，资产规模达到300亿元，信用等级力争达到AA+，争取完成融资70亿元。盘活资产，做大做强各县（区）投融资平台。全面完成农信社改制，组建农商行。积极支持商业银行、保险、基金、证券、金融中介、投资机构等在濮设立分支机构和创新拓展业务。全年新增贷款90亿元。推进企业上市融资，支持巨烽生物、班德路化学等5家企业在新三板挂牌，引导中小微企业到中原股权交易中心挂牌，鼓励发行企业债、公司债、短期融资券、中期票据等，年内实现资本市场融资120亿元以上。

培育健康养老业。加快国家级医养结合试点市建设，年底前建成综合养老服务中心等项目，开工建设濮阳高端医养中心、惠民医院幸福九号医养联合体、开发区康复养老服务中心等项目。发挥我市乒乓球等品牌优势，建设马庄桥乒乓小镇体育产业园。

3.优化供给，做优农业。年内实施重点农业项目33个，完成投资93亿元。

稳定粮食综合生产能力。加强耕地保护。实施高标准粮田“百千万”工程，开展粮食绿色高产高效创建，年内新建高标准粮田30万亩。实施渠村等大中型灌区改扩建工程，抓好小型农田水利建设，推广农业技术，提高农机化水平，藏粮于地，藏粮于技。

优化农业结构。压缩玉米种植面积，重点发展优质小麦、水稻、花生、草畜、林果和瓜菜，实现布局区域化、经营规模化、生产标准化、发展产业化。建设现代农业“三带一圈”，打造沿黄河生态农业带，重点发展特色种植、生态畜禽、水产养殖业；打造沿106国道现代特色农业示范带，重点发展日光温室、蔬菜大棚、食用菌生产等设施农业；打造沿引黄入冀补淀工程的休闲农业观光带，抓好林果、花卉种植。在市城区、县城周边规划建设生态农业园区，打造城区观光休闲农业圈。启动示范区现代农业园区规划建设。全市新建扩建提升50个现代农业示范园区。

提高农业产业化水平。培育发展新型农业经营主体，推进农村一二三产业融合发展。用足用活畜牧养殖业政策，加快推进畜牧养殖产业化发展。实施好汇源肉羊养殖加工产业园、龙丰实业食用菌、德成参业人参种植基地等项目，新培育市级农业产业化龙头企业10家，发展壮大训达油脂、家家宜米业等9个特色产业集群，不断提高农业综合效益。

4.强化融合，培育网络经济。加快互联网在制造业、服务业、农业等领域运用，培育发展互联网医疗、互联网教育等新业态。加快建设大数据智慧生态园等项目。大力发展电子商务，年内建成濮阳跨境电子商务产业园、华龙区淘金创谷电子商务产业园、台前县电商物流中心等项目，开工建设阿里巴巴濮阳运营中心、濮阳电商物流快递产业园、中大门跨境电商体验中心、网来云商（濮阳）跨境电商服务中心、范县电子商务服务中心等项目。积极支持本土电商营销濮阳特色产品。

（三）实施创新驱动发展战略

把创新摆在濮阳发展全局的核心位置，充分发挥科技创新的基础、关键和引领作用，培育发展新动能，打造产业新优势。

1.壮大创新主体。实施创新龙头企业培育工程，支持企业加大研发投入，建设一批创新型龙头企业。落实省“科技小巨人”成长行动计划，培育一批年销售额超亿元的科技型企业。年内新增高新技术企业3家、专利技术产业化示范企业5家、节能减排科技创新示范企业6家。主动对接国家、省科技发展重点，实施20项省级以上科技项目、10项市级重大科技专项。

2.筑牢创新平台。支持濮东产业集聚区创建省级高新区，力争将市经济技术开发区、濮东产业集聚区、南乐县产业集聚区纳入郑洛新国家自主创新示范区辐射区。支持企业与大专院校、科研院所建立产学研协同创新机制，争取在濮设立科技研发、中试、成果转化基地。开工建设省石化产品质检中心，新建市级及以上工程技术研究中心12家、市级重点实验室3家。实施品牌带动，建设质量强市。加快建设大学科技园、华龙区众创空间等项目，促进大众创业、万众创新。

3.汇集创新要素。创新科技投入方式，开展“科技保”“科技贷”等业务，扩大知识产权质押融资贷款规模，加快成果转化。实施人才强市战略，完善创新激励机制，突出“高精尖缺”导向，柔性引进高端人才，加快培育本地人才。强化创新能力培训，打造一支高素质企业家队伍。实施全民技能振兴工程，选树濮阳工匠，建设高技能人才队伍。

（四）强化基础支撑能力

围绕提升我市区域战略地位，补齐短板，厚植优势，创造发展新势能。

1.构建现代交通体系。抢抓机遇，加快铁路、高速公路在我市布局和建设，构建内通外联、便捷高效的综合交通体系。铁路建设，郑濮济高铁濮阳段年内完成征迁和基础工程，完成濮阳东高铁站商务区规划及站房设计工作；争取京九高铁过境设站落定；加快推进濮阳至潢川铁路前期工作，争取濮阳至开封城际铁路纳入中原城市群城际铁路规划。公路建设，加强与周边省市协调对接，共建干线公路，打通过境断头路，切实解决省界交通闭塞问题。年内，濮阳至阳新高速（城市东环）及黄河大桥、G240杨集黄河大桥及引线、S304白堽黄河大桥及引线争取开工建设，加快建设范台梁高速、台前孙口黄河大桥及引线。争取开工濮阳至卫辉高速濮阳段（城市南环），形成绕城高速。完成G106市城区段改造，拓宽改造S214，实现大型车辆绕城过境。完成G341、S215等5条国道、省道干线公路部分路段改造。改建农村公路420公里。开工建设市客运枢纽南站、综合运输西站。交通是濮阳最大的短板，我们一定要加大投入，持续发力，大干三年，彻底打破制约濮阳发展的交通瓶颈，让濮阳发展跑得更快、跑得更远。

2.强化资源能源保障能力。加快建设中部地区濮阳煤炭储配基地，煤炭储配中心年内建成投用。加快建设天然气输配中心，参股建设文23地下储气库，开工建设濮范台输气管道、鄂安沧输气管道濮阳支干线等项目。积极推进日濮洛输油管线等重大能源项目的前期工作。龙丰2×60万千瓦机组三季度建成投运。着力提高资源能源配置能力，打造国家级综合能源保障基地。

3.夯实产业发展平台。强化产业集聚区基础设施建设，完善公共研发、投融资等服务平台，增强产业承载集聚能力。全市产业集聚区完成固定资产投资1100亿元以上，实现主营业务收入1900亿元。濮阳经济技术产业集聚区、范县产业集聚区力争晋位二星级，濮东、南乐县产业集聚区力争晋位一星级。抓好服务业“两区”建设，完成基础设施投资164亿元，实现主营业务收入122亿元。华龙区特色商业区力争晋位二星级。

4.加强信息基础设施建设。完善网络基础设施，实现城乡4G网络全覆盖。实施“宽带濮阳”战略，提高宽带家庭普及率，实现城区WIFI全覆盖。加快智慧城市建设，建成智慧濮阳展示体验中心和应急指挥中心，加快濮阳“工业云”建设，启动智慧社区、智慧交通等工程。

（五）加快新型城镇化进程

准确把握我市城镇化加速期特征，充分发挥城镇化的双拉动作用，着力构建城乡统筹发展新格局。

1.推进以人为核心的城镇化。抓好国家新型城镇化综合试点市建设，认真落实“人地钱”挂钩政策，深化户籍制度改革，强化住房、医疗、教育、养老等公共服务保障，推进“三改一化”，探索农民市民化新途径。全市城镇化率提高1.6个百分点。

2.强化规划引领作用。坚持先规划、后建设。高标准完成濮阳市土地利用总体规划调整方案、濮阳市第四版城乡总体规划修编、县城总体规划修编，启动重点镇规划编制。实现中心城区、县城和重点镇控制性详规全覆盖。完善城市综合交通、路网、地下空间利用、综合管廊、绿地水系等专项规划。强化城市设计，统筹城市建筑风格，丰富文化内涵，彰显城市特色和时代风貌。

3.提升中心城市建设水平。加快城市路网建设，开工建设中原路东延、文明路北展南延、龙乡路北延、建设路东延、卫都大街东延等10条道路，构建濮—濮、濮—清和濮—东一体化发展的交通框架。打通城市干线断头路，年内建成玉门路、东濮路等16条道路。开工建设开州路与台辉高速互通立交，解决城市快进快出问题。新建改建公交港湾70个，建设市人民医院等7个地下公共停车场。完善公共服务设施，新建供水管网19公里，完成第三水厂二期工程；新建天然气供气管网3公里，实现城区供气全覆盖；新建供热管网23公里，新增集中供暖面积500万平方米；新建雨污水管网60公里。建成长庆路、龙泽大街等地下综合管廊示范工程。推进海绵城市建设，完成振兴路北段等55个市城区海绵城市建设项目。完善城区环卫设施，改造提升公厕10座。提高城市建、管、养、运水平，由城市管理向城市治理转变。加快示范区功能性综合开发，开工建设新阳光大厦、市民活动中心等项目，入驻一批重大产业项目，积极发展总部经济，尽快聚人气、见成效、出形象。

4.实施县城提质工程。抢抓省实施百城建设提质工程机遇，坚持规划先行、产业支撑，围绕提高县城承载能力这个关键，用现代城市标准建设县城。启动县城第二水源地建设，加快县城集中供暖、雨污水管网建设，完善教育、文化、医疗、体育等公共服务设施。启动实施特色小镇培育计划，突出特色产业、文化传承、休闲宜居等特征，抓好岳村杂技小镇等15个特色小城镇建设。全面实施气化濮阳工程，年内实现县（区）30个乡镇通天然气，新增天然气用户12.35万户。

5.推进美丽乡村建设。按照“五规合一”要求，科学编制新农村发展规划。坚持试点先行，对产业有基础、发展有条件、空间布局合理的村镇，先行试点建设。重点推进10个省级美丽乡村试点建设，打造100个市级美丽乡村试点和20个市级魅力村镇，发挥示范带动作用。加强环境综合整治，实施“乡村清洁工程”，6月底前各县（区）建立农村生活垃圾收运处理体系，让农村生活环境变得更洁净，让农民群众过得更舒心。

（六）全面深化改革开放

1.全面深化改革。按照“四个有利于”要求，聚焦重点领域、关键环节，实现一批重大改革新突破。

深化行政管理体制改革。认真实施权力清单、责任清单、负面清单制度，规范权力运行。加快“放、管、服”改革步伐，继续压减行政审批事项，优化审批流程，加强联审联批，清理规范中介服务，提高审批效率。整合公共资源交易服务平台，规范交易行为，实现交易公平公开。深化商事制度改革，在“五证合一”的基础上，探索实行“多证合一”，推进企业简易注册登记，推动“双随机、一公开”监管全覆盖。加强政务信息公开，促进政府信息资源共享，推行网上审批，让信息多跑路，让群众少跑腿。

深化财政性涉企资金基金化改革。发挥政府在基金管理中的主导作用，完善配套政策，创新运作模式，让基金更好地支持重点产业、重点企业发展。通过产业基金引领，促进招商引资和项目快速落地。

实施国企改革攻坚行动。分类推进市属44家国有企业改革。对自来水公司等8家优势企业，建立完善现代企业制度，支持其做强做优做大；对交运集团等12家前景较好企业，通过战略合作、兼并重组等方式整合资源，增强企业活力；对市生化公司等24家劣势企业和“僵尸企业”，依法破产，打捆托管，有序退出，做好人员安置。完成驻濮央企、省企“四供一业”移交工作。

深化农村改革。加快推进农村产权制度改革，认真解决农用地、集体建设用地、宅基地确权登记发证遗留问题。搞好产权交易服务平台建设，年内建成濮阳县、范县产权交易服务中心。加快农村集体经济股份制改革，推进农村小型水利设施产权、林地林木产权改革，赋予农民更多的财产权利。年内完成68个村股份制改革。

统筹推进医疗、医保、医药、医院联动改革。全面推进城市公立医院综合改革，深化县域综合医改，完善全民医保、药品采购、医药分离、分级诊疗制度，推广家庭医生签约服务，方便群众看病治病，减轻群众医疗负担。健全基本医保稳定可持续筹资和报销比例调整机制，推行按病种付费为主、多种付费方式结合的医保支付方式改革。

同时，深化资源型产品价格改革，建立居民用水、用电、用气阶梯价格制度。推进城市管理综合执法体制改革，6月底前完成市级改革，年底前完成县级改革。推进盐业改革，实现政企分开，盐业管理与经营分离。统筹推进文化市场综合执法改革等。

2.着力扩大开放。站位全局，主动融入国家战略，把濮阳打造成为河南东北部重要的开放支点城市。

加大招商引资力度。突出精准性，瞄准重点区域、重点产业、重点企业，挂图作战，务求突破。突出实效性，采取定点招商、专业招商、协会招商、以商招商等方式，发扬钉钉子精神，争取新落地一批重大项目。积极参加国家、省大型经贸活动，筹办好海峡两岸石油化工科技经贸交流大会，承办好第十二届豫商大会等活动。以诚待商，切实兑现招商引资优惠政策，以良好的产业生态、优质的政务服务，吸引高端项目落户濮阳。本地企业凡新上项目、技改扩能达到一定条件的，享受招商引资的同等政策。

加快开放平台建设。加快建设“无水港”，支持国检濮阳办事处拓展业务，力争海关在濮设立办事机构启动相关业务，规划建设进口保税仓和出口监管仓。依托晋豫鲁铁路和郑濮济高铁，打造豫北出海大通道。积极对接京津冀，成为环境治理、产业转移协同发展区。主动融入郑州航空港经济综合实验区、中国（郑州）跨境电子商务综合试验区、河南自由贸易试验区等重大战略，主动复制、被辐射。加强国家、省级出口基地建设。努力扩大服务贸易出口。

深化油地合作。牢固树立油地一家人、一家亲发展理念，深化双方在思想文化、战略决策、经济发展、社会管理全方位融合，进一步完善落实油地高层领导联席会议机制，加强产业发展、资源利用、基础设施和社会事业等方面的合作，携手推进中原炼化一体化等重大项目，共建国家综合能源保障基地，政府为企业建城护家园，企业为濮阳兴业拓疆土，开创油地共兴共荣、融合发展新局面。

（七）全力打好脱贫攻坚战

脱贫攻坚关乎全面建成小康社会。突出精准扶贫，“六路”并进，确保实现脱贫5.61万人。突出产业扶贫，落实好“1+5”产业扶贫政策，扶助带贫企业，培育支撑产业，加强技能培训，让更多贫困人口就近转移就业。创新金融扶贫，采取“政融保”等模式，助推农民脱贫。加大教育扶贫力度，实施教育资助政策全覆盖，绝不让一名孩子因贫失学。全面建立大病补充医疗保险制度，首先让农村贫困群众享受更多医疗保障，不再因病致贫。要移风易俗，倡导婚嫁简办，防止陋习致贫。抓好兜底扶贫，加强农村低保制度和扶贫开发政策有效衔接，应保尽保、应扶尽扶。完成黄河滩区第二批迁建试点建设任务，达到入住条件。今年，要改变基础设施不进滩的状况，建成濮范台黄河滩区公路，切实改善沿黄地区交通条件，为滩区群众修筑一条顺心路、致富路、幸福路。

（八）着力改善生态环境

坚持整治、建设、管理并重，以坚强的意志，以铁的手腕，加大工作力度，努力实现生态环境的持续改善。

1.全力抓好污染整治。持续抓好大气污染防治，强化措施，标本兼治，精准发力，不断改善空气质量，确保我市优良天数达到200天以上。启动水污染防治攻坚战，落实《濮阳市马颊河保护条例》，开展金堤河流域综合整治，消除劣V类水质，逐步恢复水体生态功能。实施城区河道截污治污、第一污水处理厂扩建等16个污染整治项目。积极推进土壤污染防治。启动实施西部化工区综合整治。以零容忍的态度严格执法监管，对环境违法行为坚决打击、决不姑息。

2.大力促进绿色发展。加快开发区、南乐县产业集聚区循环化改造。开工建设静脉产业园生活垃圾焚烧发电项目，加快建设宏业生化生物质发电等项目。实施工业节能改造、建筑节能、低碳交通、公共机构节能等工程。实施城市公共自行车租赁系统二期工程，实现市城区全覆盖。

3.加快建设水系绿系。完成濮阳市水资源利用总体规划编制，加强水资源保护和节约用水。扩大丹江水供水范围，新增供水用户8.8万户。加强全域水系建设，5月份争取引黄入冀补淀工程濮阳段全线通水，统筹沿线水系配套工程建设，实施沿河大道建设及绿化美化。开工建设引黄入鲁工程，完成澶州河开挖，拓宽改造范水河，启动清丰县、南乐县、台前县引黄调蓄水库建设。实施市城区水系规划，开挖清河，改造顺城河，开工建设前范庄、马辛庄、后河水库和马颊河、顺城河湿地，基本形成城区河道连接贯通的生态水系，让城市之水活起来、清起来。以创建国家森林城市为抓手，结合35万亩国家储备林基地建设，完善黄河、金堤河等绿色生态廊道。实施水库龙山绿化、西部林带提升工程，打造环城林系。新建提升城区公园6处，绿化提升城区道路10条，营造绿色城市空间。年内新造林8万亩，森林覆盖率达到29%。

环境是生产力，是最现实的民生，我们要撸起袖子、加油干，持续攻坚，久久为功，不久将来一定重现碧水蓝天！

（九）切实保障改善民生

实施重点民生工程，解决好涉及人民群众切身利益的突出问题，让人民群众共享改革发展成果。

1.做好就业和社保工作。强化培训，提高就业再就业能力，新增城镇就业4万人、失业再就业1.8万人、困难人员再就业5500人。完善失业预防试点政策，投入6000万元，支持困难企业职工稳定就业。全市新增农村劳动力转移就业17万人。建设25个创业示范基地，发放创业担保贷款7亿元，支持大学生、农民工等返乡创业。实施全民参保登记计划，机关事业单位养老保险实现全覆盖，适当提高企业和机关事业单位退休人员基本养老金水平。实行统一的城乡居民医保制度。落实残疾人补贴制度，市残疾人综合服务中心年内主体完工。年内建成200个农村幸福院，建成100所留守儿童之家。加强城乡社区建设，建成10个社区服务中心，创建20个示范社区，建成15个社区日间照料中心。我们要倾心尽力，让社会更和谐，让我们的老人过得更安康，让我们的孩子笑得更灿烂。

2.大力发展文化事业。全面加强精神文明建设，深入开展社会主义核心价值观教育，深化全民敬业行动，以文化人，文化铸魂。深化岗位学雷锋活动，打造濮阳志愿服务品牌。开展全民阅读活动，建设书香濮阳。优化网络舆论生态，清朗网络空间。搞好全国文明城市创建工作，确保蝉联。依托我市厚重的历史文化资源，建设濮阳华夏历史传承创新区。实施文化惠民工程，开展“舞台艺术送农民”“公益演出下基层”等活动。加强“三馆一站”建设，建设400个村级文化活动场所，市城区新增10个流动书屋，构建现代公共文化服务体系。

3.统筹发展社会事业。优先发展教育，改善162所农村中小学办学条件，市城区新建学校5所，改扩建7所，新增学位8000个。启动学前教育三年行动计划，新改扩建公办幼儿园38所，扶持普惠性民办幼儿园42所。全年资助困难学生60万人次。支持濮阳职业技术学院开展联合办学，建设3D体验学院。濮阳医学高等专科学校力争上半年获批挂牌。加快市体校迁建。推进实施奥林匹克公园综合提升，建设200处农村健身场所，广泛开展全民健身活动。举办乒乓球、重竞技等全国性赛事。

贯彻全省卫生与健康大会精神，把人民健康放在重要地位，树立大卫生、大健康理念，逐步由治病向全方位、全周期的健康服务转变。以县级医院综合改革为抓手，实施“369”人才工程，加强县、乡、社区医疗标准化建设，实现以县带乡、县乡一体化医疗服务。巩固国家卫生城市创建成果，积极创建国家健康城市示范市，实施健康细胞工程。建成投用市人民医院病房楼等项目，开工建设市妇幼保健院妇儿保健综合楼、示范区妇儿医院等项目。实施新生儿代谢病基因筛查，为5万农村妇女免费实施“两癌”筛查。

4.维护社会大局稳定。严格落实安全生产责任制，坚决遏制重特大事故发生。以创建国家食品安全城市为统领，加强监管，确保群众饮食用药安全。加强平安濮阳建设，新改建提升1247个警务工作站，新建30个乡镇600个村基层综治中心。持续推进社会治理创新，构建立体化社会治安防控体系，严厉打击违法犯罪活动。完善应急管理体系，提升应急处置能力。做好信访稳定工作，完善矛盾纠纷化解处理机制，建设和谐濮阳。

加强国防教育和国防后备力量建设，支持驻濮解放军、武警消防部队、预备役部队、民兵和人民防空建设，深入开展双拥共建，巩固军政军民团结。做好民族宗教工作。统筹做好统计、发展研究、外事侨务、机关事务、驻外联络、公积金管理、残疾人服务、史志、档案、防震减灾、气象、邮政、通信等工作。

四、切实强化政府自身建设

加强法治政府、创新政府、廉洁政府和服务型政府建设，提高执行力和公信力，努力做好政府工作。

增强“四个意识”，坚定政治方向。始终在思想上政治上行动上同以习近平同志为核心的党中央保持高度一致，坚决维护党中央权威，坚守政治信仰，站稳政治立场。切实增强政治意识、大局意识、核心意识、看齐意识，向党的理论和路线方针政策看齐，围绕核心聚力，在大局中明正方向，在政治上考量行动。严守政治纪律和政治规矩，不折不扣贯彻落实好党中央、国务院和省委、省政府、市委作出的决策部署。

坚持依法行政，规范权力运行。牢固树立法治理念，把依法行政、依法办事原则贯穿到政府工作全过程，尊法学法守法用法，加快建设法治政府。提高法治素质和法治能力，自觉运用法治思维和法治方式深化改革、推动发展、化解矛盾、维护稳定。加强服务型行政执法和重点领域综合执法，落实行政执法责任制。严格执法监督，做好行政复议和行政应诉，创造公正公平的法治环境。做好“七五”普法工作。自觉接受人大法律监督、政协民主监督、新闻媒体和社会公众监督，认真落实人大决议，办理好人大议案、代表建议和政协提案。广泛听取民主党派、工商联、无党派人士和各人民团体意见。坚持科学民主决策，严格决策程序，建立重大决策终身责任追究制度和责任倒查机制。扎紧织牢制度笼子，让权力在阳光下规范运行。

完善体制机制，切实提升效能。建立分工负责、协调配合、高效运作的政府运行机制。强化目标责任、执行监督、绩效考核，提高行政执行力。完善领导分包、观摩考评、现场办公、台账管理、项目调度等工作机制，加强督导督查，狠抓工作落实。强化监察、审计监督，严格执行首问负责、限时办结制，提升行政效能，创造一流的政务环境。建立容错纠错机制，营造支持改革创新、敢于担当负责的浓厚氛围，为改革创新者撑腰，为敢担当者担当。

着力提升素质，持续转变作风。巩固提升“两学一做”学习教育成果，建设学习型政府，持续提高政治素质和业务素质。全面整治庸政懒政怠政，坚决纠正不作为、乱作为。勇于打破传统路径依赖，以新理念、新思路谋划推动工作、破解发展难题。坚持求真务实，科学谋划、统筹运作，定一件干一件、干一件成一件，以实干干实回报人民的期望。

践行为民宗旨，做到廉洁从政。坚持以人民为中心发展思想，把维护、实现好人民群众根本利益作为政府工作的出发点和落脚点，深入基层，听民意、察民情、解民忧。深入贯彻落实中央八项规定精神，严格执行新修订的两个《准则》和三个《条例》，持续强化正风肃纪，保持惩治腐败的高压态势。落实党风廉政建设和惩防体系建设的主体责任，严格执行廉洁自律各项规定，清白做人、干净做事，不忘初心、当好公仆。

各位代表！新一年、新希望，新征程、新答卷。神圣使命鼓舞我们砥砺奋进；人民期盼鞭策我们勇往直前。让我们紧密团结在以习近平同志为核心的党中央周围，在省委、省政府和市委的坚强领导下，凝心聚力，埋头苦干，为全面建成小康社会、加快建设富裕文明和谐美丽新濮阳做出新的更大贡献！
